# Role of YAP Signaling in Regulation of Programmed Cell Death and Drug Resistance in Cancer

**DOI:** 10.7150/ijbs.83586

**Published:** 2024-01-01

**Authors:** Wei Zhou, Adrian Lim, Mouad Edderkaoui, Arsen Osipov, Heshui Wu, Qiang Wang, Stephen Pandol

**Affiliations:** 1 Department of Medicine, Cedars-Sinai Medical Center, Los Angeles, California, USA.; 2 Department of Pancreatic Surgery, Union Hospital, Tongji Medical College, Huazhong University of Science and Technology, Wuhan, China.

**Keywords:** Programmed cell death, YAP/TAZ, Apoptosis, Ferroptosis, Autophagy, Drug resistance

## Abstract

Although recent advances in cancer treatment significantly improved the prognosis of patients, drug resistance remains a major challenge. Targeting programmed cell death is a major approach of antitumor drug development. Deregulation of programmed cell death (PCD) contributes to resistance to a variety of cancer therapeutics. Yes-associated protein (YAP) and its paralog TAZ, the main downstream effectors of the Hippo pathway, are aberrantly activated in a variety of human malignancies. The Hippo-YAP pathway, which was originally identified in Drosophila, is well conserved in humans and plays a defining role in regulation of cell fate, tissue growth and regeneration. Activation of YAP signaling has emerged as a key mechanism involved in promoting cancer cell proliferation, metastasis, and drug resistance. Understanding the role of YAP/TAZ signaling network in PCD and drug resistance could facilitate the development of effective strategies for cancer therapeutics.

## Current landscape of cancer therapy and challenge

Surgery combined with chemotherapy or radiotherapy are currently the first-line treatment modalities for localized tumors. However, many patients suffer from tumor recurrence and development of acquired resistance despite success in initial treatment 1, 2. Most patients with advanced solid tumors, such as hepatocellular carcinoma, pancreatic adenocarcinoma and glioblastoma, show intrinsic resistance to chemotherapy and other antitumor agents at the initial stage 3-5. Drug resistance in cancer is multifaceted and can be mediated by mechanisms that impact on drug availability, cell proliferation and response to DNA damage or metabolic pathways. Deregulation of cell death signaling represents an important mechanism of drug resistance because most of the anti-tumor therapy agents aim to trigger programmed cell death 6. Thus, understanding the signaling pathways involved in regulating PCD and drug resistance can provide insights into development of new therapeutic targets.

## The Hippo-YAP signaling pathway

The Hippo-YAP signaling plays an important role in aspects of malignant transformation, including cell proliferation, tumor progression, metastasis, and drug resistance 7-10. YAP and its paralog TAZ are the main downstream effectors of the Hippo-YAP pathway and act as a transcriptional coactivator 11-13. The YAP signaling can translocate into the nucleus and mediates gene transcription by binding to transcription factors, such as the TEA domain family (TEAD) proteins 12. YAP exhibits oncogenic activities 14, 15 and is upregulated in most solid tumors 16-22. YAP signaling target genes participate in regulation of development, cell proliferation, migration and survival 23-27.

YAP signaling is responsive to intercellular adhesion, cell density, and mechanical stiffness of the extracellular matrix 7-10. YAP can be negatively regulated by a cascade of phosphorylation events that are mediated by mammalian Ste20-like kinases1/2 (MST1/2, the mammalian homolog of Hippo) and large tumor suppressor 1/2 (LATS1/2) 11, 28, 29. The scaffold proteins of adherens junctions, such as NF2 or KIBRA/WWC1, can recruit MST and LATS kinases to the plasma membrane and mediates their activation 30-33. The kinase activity of MST1/2 can be activated by binding to the Salvador Family WW Domain Containing Protein 1 (SAV1), a scaffold protein that also forms a complex with LATS1/2^11^, ^28^, ^29^, ^34^. The Ras Association Domain Family Members (RASSFs) can also associate with MST1 and enhance its kinase activity 35-37.MST1/2 subsequently activates LATS1/2 by phosphorylating LATS1/2 and its regulatory protein Mps one binder kinase activator-1 (MOB-1) 38, 39. In parallel to MST1/2, the MAP4K family kinases can also phosphorylate and activate LATS1/2 40-42. The activated LATS1/2 then phosphorylates YAP/TAZ 43, 44. The phosphorylated YAP can associate with the 14-3-3 proteins and is rendered transcriptionally inactive due to retention in the cytoplasm 45, 46. Alternatively, the phosphorylated forms of the YAP/TAZ proteins can also be primed for β-TrCP-mediated ubiquitination and degradation 13, 43, 47. In addition, YAP can be inactivated by binding to a series of proteins, such as angiomotin, Protein Tyrosine Phosphatase Non-Receptor Type 14 (PTPN14) and tight junction protein zonula occludens 48-51. Conversely, YAP can be activated by G-protein coupled receptors or the mevalonate pathway through rho GTPase signaling 52-55. Epithelial cell transforming 2 (ECT2), a guanine nucleotide exchange factor for Rho-like GTPases that activates Rho signaling, can positively regulate YAP function and is reciprocally regulated by YAP 56. The SRC family tyrosine kinases and the c-ABL kinase have also been reported to promote YAP signaling by tyrosine phosphorylation of YAP 57-59.

In addition to phosphorylation, YAP signaling can be regulated by other forms of post-transcriptional modifications. For example, YAP can be methylated by the SET Domain Containing 1A** (**SET1A) methyltransferase complex, which promotes its oncogenic activities by blocking nuclear export 60. YAP can also be modified by O-GlcNAcylation that prevents its phosphorylation by LATS1, leading to nuclear localization and enhanced tumorigenic functions 61. Moreover, O-GlcNAcylation of LAST2 has been reported to cause YAP/TAZ activation and promote tumor growth 62. Acetylation of LAST1 inhibits YAP phosphorylation and degradation and promotes cancer cell invasion and growth 63. Thus, post-transcriptional modification is an important mechanism in regulating YAP signaling.

## YAP signaling activation and Drug Resistance

A growing number of studies reveal that activation of YAP signaling contributes to resistance to chemotherapy, targeted therapy and immunotherapy.

### (1) YAP signaling and Chemotherapy

DNA-damaging agents, such as doxorubicin, irinotecan, oxaliplatin, and cisplatin, aim at DNA replication as a target to induce cytotoxic effects and are widely used in the clinics. YAP signaling is closely linked to the resistance of DNA-damaging agents. YAP is a key regulator of doxorubicin resistance in thyroid cancer and is regulated by tripartite motif-containing protein 11 (TRIM11) 64. Overexpression of YAP confers resistance to doxorubicin by regulating bcl-xl 65. Activation of YAP also promotes doxorubicin chemoresistance in cholangiocarcinoma and osteosarcoma cells 20, 66. Inhibition of YAP enhances oxaliplatin and irinotecan sensitivity 67, 68. YAP activation induces cisplatin resistance in small cell lung cancer (SCLC) cells 69, whereas knockdown of YAP increases the sensitivity of cisplatin in ovarian cancer cells 48, 70. In addition, overexpression of TAZ is involved in regulation of cisplatin resistance of cervical, gastric, lung and ovarian cancer 22, 71-73.

Agents inhibiting metabolic pathways represent another major class of chemotherapy agents that can sabotage DNA or RNA synthesis and inhibit cell division and survival. Gemcitabine and 5-fluorouracil (5-FU) are cytidine and uracil nucleoside analogues, respectively, which are widely used for the treatment of multiple tumors. Knockdown of YAP enhances gemcitabine sensitivity 74. In addition, high levels of YAP are associated with poor survival in patients following 5-FU treatment, which is accompanied with an increase of M2 polarization of macrophage in the tumor 75. However, a recent study reported that overexpression of an activating mutant form of YAP appears to enhance cancer cell sensitivity to gemcitabine and 5-FU, by reducing drug efflux 76. It should be noted that these findings remain to be corroborated with knockdown studies.

Anti-microtubule agents block mitosis by interfering with microtubules dynamics and induce apoptosis. Examples of classical anti-microtubule agents include taxanes (paclitaxel and docetaxel), which are widely used for the treatment of breast, ovarian, gastric, pancreatic and colorectal cancer 77. YAP has been reported to confer resistance to paclitaxel in cancer cells 78, 79. Similarly, TAZ and TAZ/TEAD-mediated expression of Cyr61 and CTGF are also vital in paclitaxel response 21, 80. Moreover, down-regulation of YAP has been found to enhance sensitivity to docetaxel 81. The mechanism by which YAP signaling modulates resistance to taxanes may involve YAP- and TEAD-regulated expression of ATP Binding Cassette Subfamily B Member 1 (ABCB1), which encodes the multidrug resistance protein 1 and is implicated in paclitaxel resistance 82. In addition, YAP mediates the expression of an array of mitotic genes and deregulation YAP signaling can lead to aberrant mitotic checkpoint control 83, 84. The mitotic regulator cyclin-dependent kinase 1 (CDK1) phosphorylates YAP in response to paclitaxel-induced G_2_/M arrest 85. But its role in drug resistance remains to be clarified.

Thus, an increasing body of evidence indicate that activation of YAP signaling results in chemoresistance and inhibition of this pathway may enhance cancer cell sensitivity to chemotherapeutic drugs. Targeting YAP signaling pathway represents a potential strategy to overcome chemotherapy resistance. Indeed, the combination of YAP inhibitor with chemotherapy has shown increased efficacy in chemo-resistant tumors 86, 87.

### (2) YAP signaling and Targeted Therapy

Targeted therapy is designed to block molecules and pathways that are vital for cancer cell proliferation, survival, invasion and metastasis and rewrote the paradigm of leukemia treatment 88. Targeted therapy for epidermal growth factor receptor (EGFR), human epidermal growth factor receptor 2 (HER-2), vascular endothelial growth factor receptor (VEGFR), and BRAF have been developed for clinical use 88. In this section, we will briefly discuss how YAP signaling regulates targeted therapies.

BRAF, a serine/threonine protein kinase of the RAF kinase family, is a key regulator of the MAP kinase signal transduction pathway. BRAF gene mutations occur in a large percentage of cancers, including approximately 50% of melanomas, 20% to 40% of thyroid cancers, and 10% of colorectal cancers 89. Although BRAF inhibitors have shown benefit in melanoma patients with the oncogenic BRAF^V600E^ mutant, acquired drug resistance remains a significant obstacle 90. NF-2, a negative regulator of YAP signaling, was identified as a gene associated with cancer cell sensitivity to BRAF inhibitor, which indicates that YAP signaling could participate in BRAF inhibitor resistance 91. In a separate study, activation of YAP was shown to induce resistance to BRAF inhibitor in melanoma cells through the actin dynamic regulator testis associated actin remodeling kinase 1 (TESK1) 92. In addition, YAP confers immune evasion in BRAF inhibitor resistant melanoma cells by promoting PD-L1 expression, which can be targeted by immune checkpoint therapy 93. This finding suggests that the combination of BRAF inhibitor with immunotherapy may represent a viable approach to treat BRAF inhibitor resistant melanoma.

EGFR is a receptor tyrosine kinase that is frequently mutated in many tumors 94. Small-molecule tyrosine kinase inhibitors (TKIs) for EGFR have shown efficacy in treatment of EGFR-mutated tumors 95. However, resistance remains a problem for clinicians. Numerous studies have indicated that activation of YAP/TAZ is widely associated with in EGFR TKI resistance 96-98. YAP regulates epithelial-to-mesenchymal transition (EMT)-induced resistance to EGFR TKI in non-small cell lung cancer (NSCLC) via FOXM1/SAC pathway 99. YAP could also mediate EGFR TKI-resistant through upregulation of AXL receptor tyrosine kinase 100 or the autophagy mediator p62^101^. Combination of YAP inhibitor and EGFR TKI improved response in EGFR inhibitor resistant NSCLC 102.

YAP signaling is also implicated in resistance to targeted therapies for HER-2, MEK, RAS, ALK and BET inhibitors. YAP1 dephosphorylation and TEAD2 overexpression are closely related to trastuzumab resistance by regulating cytokines like CCL5 in HER-2 positive breast cancer cell lines 103. YAP deletion sensitizes the MEK inhibitor trametinib by depletion of MYC/MYCN and E2F transcriptional output in neuroblastoma cells 104. Dasatinib can enhance the antitumor effect of trametinib in KRAS-mutant cancer models by inhibiting the expression of TAZ protein 105. YAP activation promotes resistance to ALK-TKI through induction of p21 expression 106. TAZ nuclear localization and transcriptional activity induces resistance to inhibitors of BET family proteins 107. In summary, activation of YAP/TAZ signaling contributes to the resistance to an array of targeted therapy agents in various tumors. Targeting YAP signaling may improve the outcomes of targeted therapy.

### (3) YAP signaling and Immunotherapy

The recent success of using immune checkpoint inhibitors for the treatment of certain human cancers represents a breakthrough in immunotherapy. Immune checkpoint receptors play a critical role in the maintenance of immune homeostasis. The classic immune checkpoint receptors include PD-1, CTLA-4, and TIGIT. The engagement of the immune checkpoint receptors can result in anergy of CD8^+^ T cells and enhance tumorigenesis and invasiveness of tumors. Immune checkpoint inhibitors remove inhibitory signals of T-cell activation, which enables tumor-reactive T cells to mount an effective antitumor response 108, 109. The anti-PD-1/PD-L1 agents, the most used immune checkpoint inhibitors, has shown promising outcomes in the treatment of certain cancer types, significantly extending the overall survival of patients 110. Monoclonal antibodies against PD-1/PD-L1 or CTLA-4 have shown promising efficacy in certain tumors 111-113. Emerging evidence indicates an important role for YAP signaling in modulating anti-PD-1/PD-L1 immunotherapy. PD-L1 is a direct transcriptional target of YAP signaling, knockdown of YAP inhibits expression of PD-L1 and reverses resistance to EGFR-TKI 114. TAZ also upregulates PD-L1 expression in pancreatic cancer, leading to immune evasion and immunotherapy resistance 115. Activation of YAP-mediated transcriptional hubs in the nuclei is associated with resistance of anti-PD-1 in a mouse model of lung cancer cells, and inhibition of YAP can enhance the efficacy of anti-PD-1 therapy 116. In addition, YAP signaling in cancer cells can facilitate recruitment of macrophages or myeloid-derived suppressor cells (MDSC) to the tumor microenvironment by regulating CXCL5, IL-6 and Csf1-3, and inhibition of YAP-mediated immune cell infiltration impairs tumor growth 117, 118. These findings indicate that targeting YAP signaling may improve the efficacy of immunotherapy.

## YAP signaling and Programmed Cell Death

Apoptosis, ferroptosis and other forms of PCD are associated with cancer drug resistance 119, 120. Recent studies discovered that YAP signaling plays an important role in the regulation of PCD in cancer 121. Understanding the relationship between YAP signaling and PCD can assist the development of more effective cancer therapeutic strategies.

## YAP signaling and Apoptosis

Apoptosis is a major cancer cell response to most therapeutic drugs. Dysregulation of apoptosis contributes to tumorigenesis and drug resistance 119. YAP signaling participates in the regulation of apoptosis and inhibition of YAP signaling can promote apoptosis via multiple pathways. Inhibition of YAP signaling can promote apoptosis in multiple pathways. Knockdown of YAP and TAZ can enhance apoptosis under hypoxic condition 122. YAP appears to modulate cancer cell susceptibility to apoptosis triggered by an ER stress inducing agent 123. Knockdown of YAP can sensitize colon cancer cells to inhibitors of the MAPK pathway, which may involve YAP-mediated expression of CDK6 124. YAP is implicated in playing a role in determining the switch between apoptotic and survival pathways following activation of G protein-coupled bile acid receptor (GPBAR) signaling 125. Inhibition of YAP increases cancer cell apoptosis induced by genotoxic agents 126. Knockdown of YAP enhances apoptosis in cancer cells treated with the Abl and Src family kinase inhibitor bosutinib, which is associated with mitochondrial fragmentation and reactive oxygen species (ROS) accumulation 127. Indeed, knockdown of YAP increases mitochondrial fission via JNK-Drp1, which can lead to apoptosis 128.

Activation of YAP can inhibit apoptosis by mediating the pro-apoptotic function of nuclear receptor 4A1 (NR4A1) 129. Knockdown of YAP in cancer cells increases the levels of ER stress and apoptosis 18. Ras association domain family member 4 (RASSF4) enhances apoptosis by decreasing YAP-regulated bcl-2 expression 130. Knockdown of YAP can induce apoptosis by reducing SIRT1- and Mfn2-mediated mitophagy 131. The YAP/TEAD4 complex can inhibit apoptosis and promote cancer progression by activating kinesin family member 4A (KIF4A) expression 132. Overexpression of YAP promotes proliferation and suppress apoptosis via increase of bcl-2 133. YAP knockdown sensitizes bladder cancer cells to cisplatin-induced apoptosis, which is accompanied with downregulation of surviving 134. A recent study showed that YAP plays a vital role in evasion of apoptosis by mediating cancer cell dormancy, which involves recruitment of the EMT transcriptional factor SLUG and suppression of the expression of the pro-apoptotic protein bcl-2-modifying factor (BMF) 135.

Further investigation of the precise mechanisms regarding the regulation of apoptosis by YAP signaling may contribute to the discovery of potential therapeutic targets.

## YAP signaling and Ferroptosis

Ferroptosis is a biochemically and morphologically distinct form of PCD characterized by iron-dependent lipid peroxidation and compromise of cell membrane integrity 136. Lipid peroxidation is a process under which oxidants such as free radicals and intracellular ROS attack lipids especially polyunsaturated fatty acids, leads to lipid peroxidation and cell death. Imbalance of iron redox ability leads to the production of oxygen free radicals and damages various cellular components, eventually induces ferroptosis. Cancer cells have developed many defense mechanisms to prevent lipid peroxidation. The most well-known is the glutathione peroxidase 4 (GPX4)-glutathione (GSH) system. GPX4 can reduce peroxidized lipids to their corresponding alcohols by binding to its cofactor GSH 137. Ferroptosis is associated with multiple diseases, such as cancer, inflammation, heart injury and sepsis 138-141. A growing number of studies indicates that YAP signaling modulates ferroptosis by regulating expression of genes involved in keeping the balance of intracellular ROS and lipid peroxidation.

YAP can modulate iron concentration through the transcriptional regulation of transferrin receptor (TFRC) 142. Activation of YAP also confers sensitivity to ferroptosis via regulation of the arachidonate lipoxygenase 3 (ALOXE3) 143, which promotes lipid peroxidation and ferroptosis 144, or by upregulating multiple regulators of ferroptosis, particularly TRFC and acyl-CoA synthetase long chain family member 4 (ACSL4) 145. TAZ mediates ferroptosis through indirectly regulating the expression of the ROS-generating nicotinamide adenine dinucleotide phosphate oxidases (NOX) through angiopoietin-like 4 (ANGPLT4)-NOX2 and epithelial membrane protein 1 (EMP1)-NOX4 axis 146, 147. YAP/p53 axis is required for lipid peroxidation and ferroptosis induced by cytoglobin, a heme-binding protein that mediates redox homeostasis in cells 148.

However, in a separate line of studies, YAP appears to have anti-ferroptosis effect. YAP/TAZ mediate the expression of solute carrier family 7 member 11 (SLC7A11), a subunit of the cystine/glutamate transporter that is important for maintaining intracellular cysteine and glutathione storage, and thus contributes to resistance to ferroptosis 149, 150. Induction of ferroptosis by inhibition of the cystine/glutamate transporter system by erastin is accompanied with glutamate-induced O-GlcNAcylation and down regulation of YAP, and ectopic expression of a mutant form of YAP that cannot undergo O-GlcNAcylation reduces sensitivity to ferroptosis 151. Moreover, YAP can also protect cells from ferroptosis by suppressing the expression of ferritin light chain, a major protein important for storing intracellular iron 152.

In summary, YAP signaling can regulate genes involved in different aspects of lipid peroxidation. The overall effect of YAP disruption on ferroptosis may depends on the genetic background of the cell types or the metabolic environment.

## YAP signaling and Autophagy

Autophagy is a mechanism by which cells adapt to physiological or pathological changes by degrading and recycling parts of the cell in a lysosome-dependent manner. Autophagy is vital in maintaining organismal homeostasis 153, 154 and dysfunction of autophagy is implicated in multiple diseases 155-157. The molecular mechanism of autophagy involves several autophagy-associated proteins (ATG). Various stimuli, such as nutrient deficiencies and hypoxia, can leads to the formation of phagocytic vesicles, a step regulated by two protein complexes. One is the Vps34 complex containing Vps34, ATG6, ATG14 and Vps15. The other is the unc-51 like autophagy activating kinase (ULK1)/ATG1 complex, which is an important positive regulator of autophagosome formation 158. Autophagy is involved in aspects of tumorigenesis, including cancer cell survival, invasion and immune response 159-162. Recent studies have shown complex interactions between YAP signaling and autophagic pathways 163.

YAP signaling promotes autophagy in most tumors. YAP is required for lncRNA-ATB induced autophagy 164. YAP activates autophagy by promoting ATG5 transcription, while autophagy in turn negatively regulates YAP through autophagic degradation 165. Disruption of autophagy by genetic deletion of ATG7 in the hepatocytes leads to upregulation of YAP and malignant transformation in the liver, which can be reversed by concurrent deletion of YAP 166. Silencing of YAP leads to impaired autophagy, which enhances cisplatin sensitivity in cancer cells 70. Proto-oncogene SKIL could induce TAZ-dependent autophagy in NSCLC cell lines 167. Cisplatin-induced autophagy can activate YAP by decreasing phosphorylation 168. YAP promotes autophagy and tumor progression in glioblastoma through upregulation of high mobility group box 1 (HMGB1) 17.

Contrary to the above-mentioned studies, YAP signaling has also been shown to inhibit autophagy. YAP can suppress autophagy in sarcoma 169. Autophagy induced by depletion of TAZ could inhibit migration and invasion 170. Blockade of YAP induces autophagy‑related cell death and confers sensitivity of chemotherapy 19. YAP inhibits autophagy through the suppression of phosphatase and tensin homolog (PTEN) and activation of the AKT/mTOR pathway 171. Moreover, YAP inhibits autophagy by upregulating expression of bcl-2 172.

In summary, YAP signaling appears to play opposing roles in regulation of autophagy, which may be dependent on the genetic background of the tumor. The mechanisms involving in the relationship between YAP signaling and autophagy needs further exploration.

## YAP signaling and Pyroptosis

Pyroptosis, also known as inflammatory necrosis, is a type of PCD characterized by Gasdemin-mediated membrane rupture and release of inflammatory molecules 173. Pyroptosis is usually initiated in response to viral and bacterial infections, accompanied by activation of the inflammasome and secretion of pro-inflammatory cytokines 174. Pyroptosis facilitates inflammatory microenvironment, which promotes carcinogenesis and metastasis 175. The role of pyroptosis in tumor development is complicated. In the initiation stage, pyroptosis can promote tumor development through inflammasome or the release of pro-inflammatory cytokines, such as interleukin-1β and IL-18 176, 177. In later stages, the inhibition of pyroptosis may promote tumor progression 178.

Pyroptosis is involved in the regulation of drug resistance in cancer. Downregulation of Gasdermin E (GSDME), a key regulator of pyroptosis, confers retinoblastoma cells resistance to chemotherapy 179. Bioinformatics analysis reveals that four regulatory genes of pyroptosis are closely related to temozolomide resistance in glioma 180. Caspase-1/GSDMD dependent pyroptosis is involved in cisplatin resistance of NSCLC cells 181. Pyroptosis induced by STAT-3β enhances cisplatin sensitivity in esophageal squamous carcinoma cells 182. In addition, pyroptosis-related gene signature has shown promise in predicting the efficacy of immunotherapy in multiple cancer types 183. Pyroptosis improves the sensitivity of immunotherapy by remodeling tumor microenvironment 184.

Existing research regarding YAP signaling on the regulation of pyroptosis is mostly focused on non-cancerous diseases like infection, inflammation and diabetes 185-187 and only a few studies explored the role of YAP signaling in cancerous pyroptosis. Inactivation of YAP switch chemotherapy induced cell death from apoptosis to pyroptosis through upregulating the expression of GSDME 69. A recent study showed that MST1 can promote ROS-induced pyroptosis, which is accompanied by inactivation of YAP via phosphorylation and results in suppression of tumor cell proliferation and invasion 188.

## Conclusion and Future Perspectives

In summary, YAP signaling is engaged in the regulation of multiple forms of programmed cell death, including apoptosis, ferroptosis, autophagy, and pyroptosis. Activation of YAP/TAZ contributes to resistance to a variety of tumor therapeutic modalities, such as chemotherapy, targeted therapy and immunotherapy. Verteporfin (VP), originally used for treating fundus macular degeneration, possesses potency of effectively YAP inhibition 189. Several studies indicate that VP could increase sensitivity to targeted or chemotherapy drugs by inhibition of YAP 190-194. Clinical trials using VP for the treatment of pancreatic cancer are underway 195. As such, VP has the potential to become an anti-tumor agent of multiple tumor types in the future. More recently, a pan-TEAD inhibitor has been developed and showed activity in blocking YAP signaling and overcoming KRAS G12C inhibitor resistance 196. Moreover, small molecule inhibitors of TEAD auto-palmitoylation have also been reported to exhibit potency to inhibit NF2-deficient Mesothelioma 197. Similarly, K-975, a TEAD inhibitor, can inhibit the proliferation of malignant pleural mesothelioma (MPM) cell lines and provide significant survival benefit in MPM xenograft model 198. In addition, several other YAP/TEAD inhibitors are currently tested in the preclinical and clinical research stages, and may provide more drug choices for YAP/TEAD based anti-tumor therapy in the future 199. The advancement of our understanding in YAP or YAP-mediated signaling events in cancer drug resistance may lead to development of new therapeutic regimen for cancer.

## Figures and Tables

**Figure 1:**
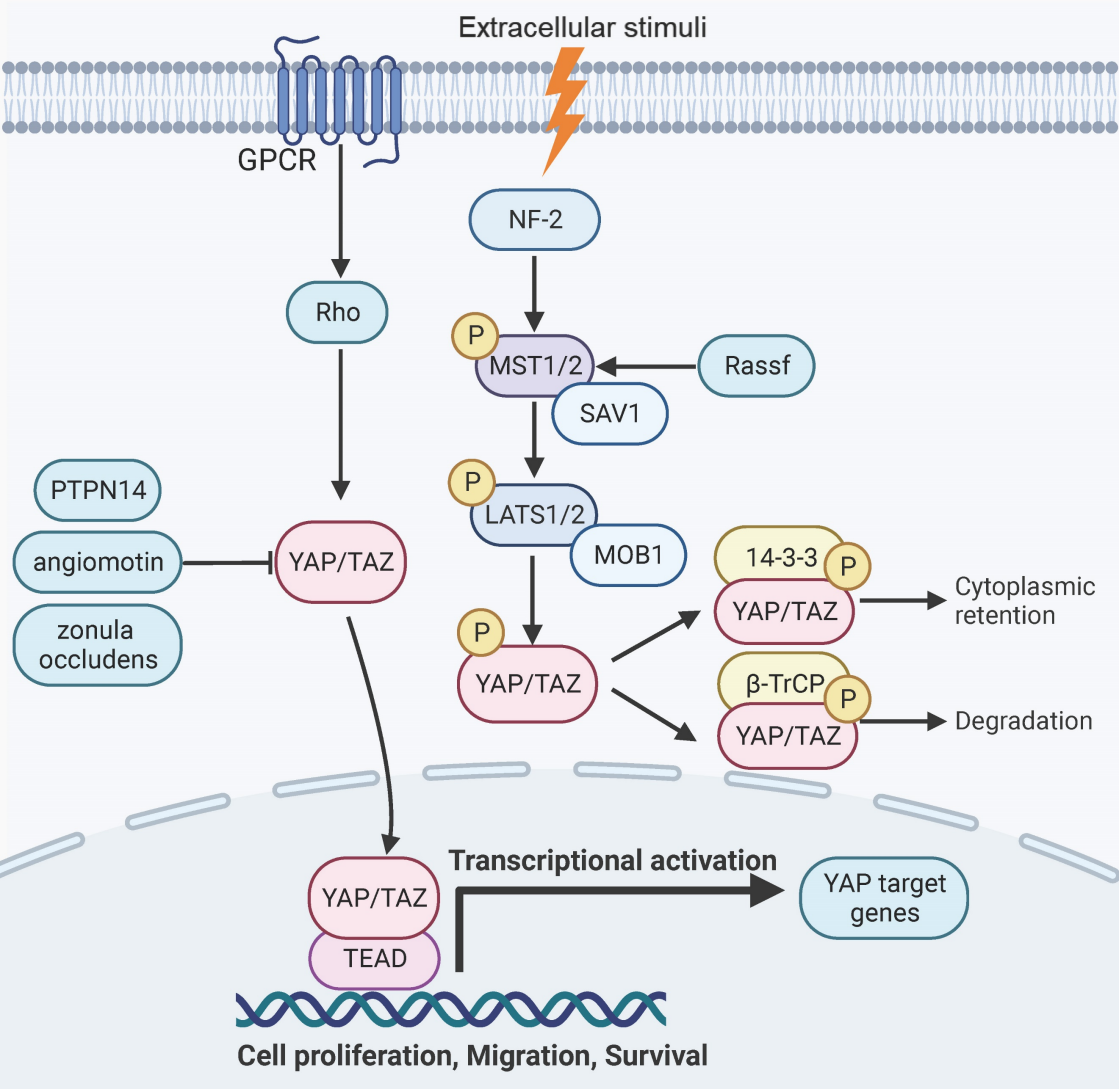
** Hippo-YAP pathway signaling and function.** YAP signaling is negatively regulated through a cascade of phosphorylation events mediated by MST1/2 and LATS1/2, which leads to phosphorylation of YAP and subsequent proteasomal degradation or retention in the cytoplasm. Alternatively, YAP can be inactivated by binding to angiomotin, PTPN14 and zonula occludens. Signaling events triggered by GPCR and Rho can activate YAP by inducing its nuclear translocation. YAP target genes are involved in aspects of organ development, regeneration, and malignant transformation.

**Figure 2:**
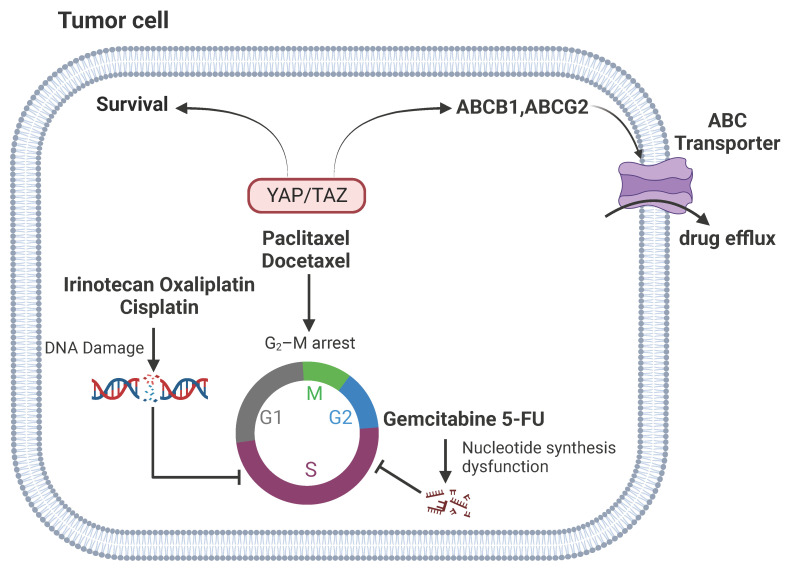
** YAP signaling and chemotherapy.** YAP/TAZ-mediated tumor chemoresistance by increasing survival in DNA damage and cell cycle arrest and reducing drug efflux. Anti-microtubule chemotherapeutic agents can induce G_2_/M cell cycle blockade. YAP signaling regulates their resistance through pathways such as CTGF/Cyr61, ABCB1, and CDKs. YAP signaling modulates resistance to DNA damage agents through TRIM11, p53, IL-8 pathways. Gemcitabine and 5-FU affect DNA and RNA synthesis in tumor cells. YAP signaling regulates their resistance through M2 polarization.

**Figure 3:**
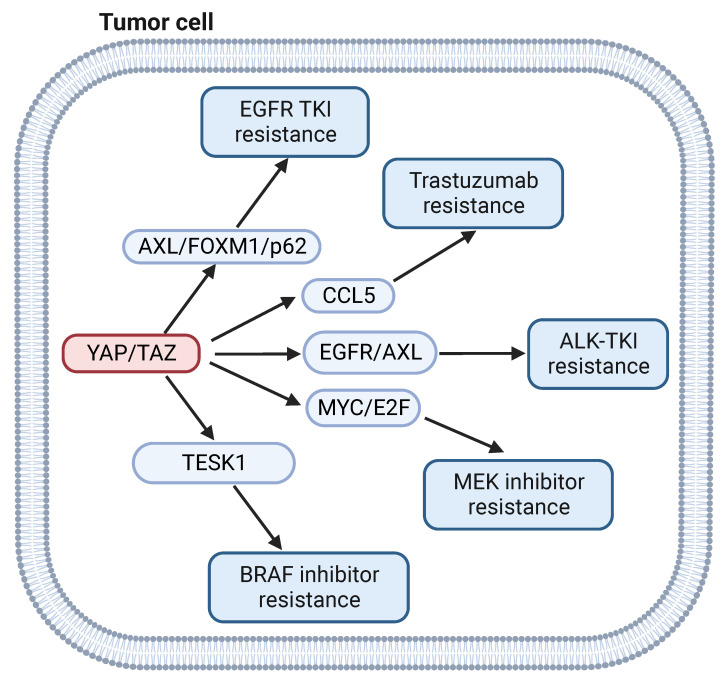
** YAP signaling and targeted therapy.** YAP signaling drives resistance of BRAF inhibitors by regulating TESK1. YAP signaling mediates EGFR TKI resistance by via FOXM1/p62. YAP signaling mediates Trastuzumab, ALK TKI and MEK inhibitors resistance by via CCL5, EGFR/AXL and MYC/E2F, respectively.

**Figure 4:**
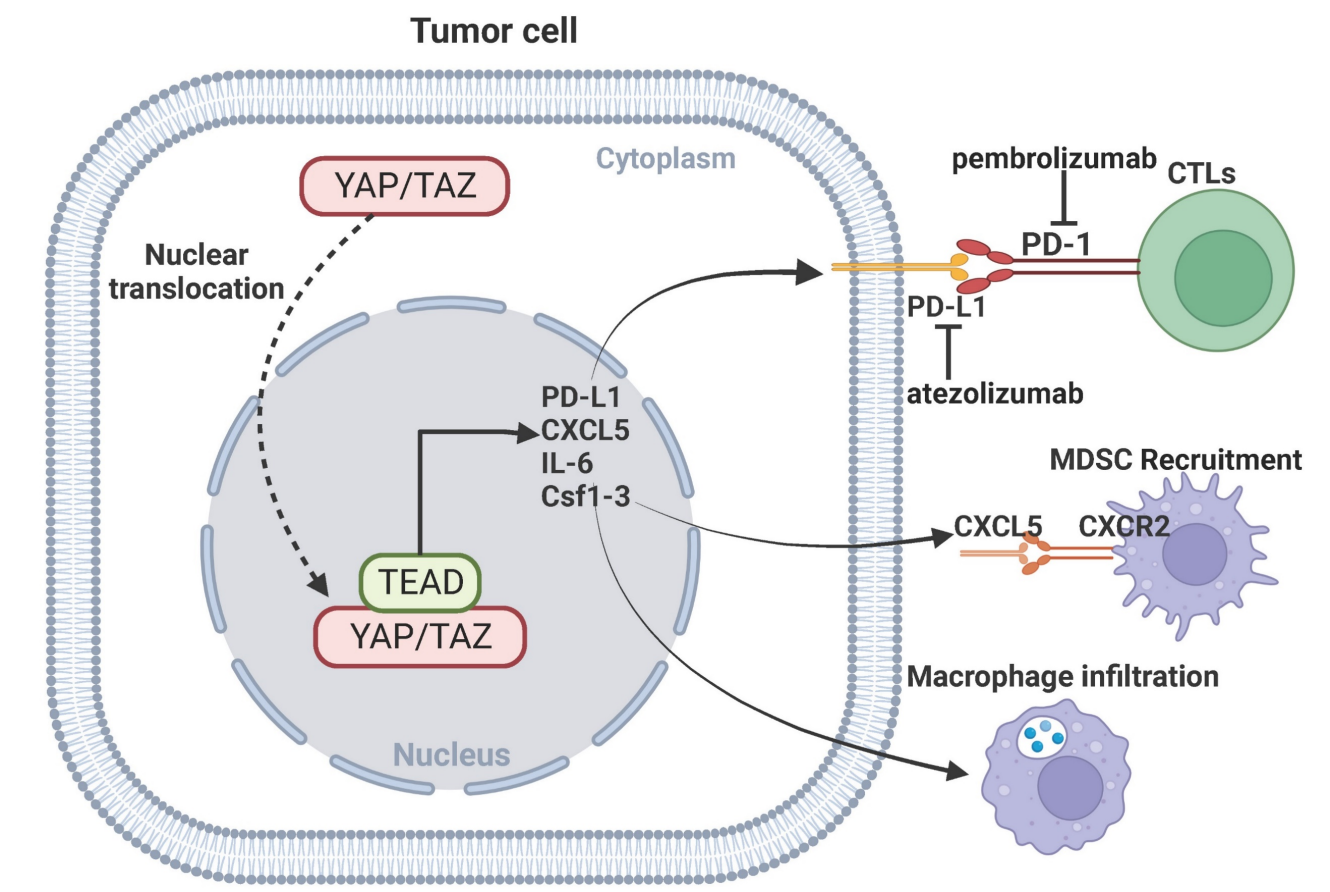
** YAP signaling and immunotherapy.** YAP mediates immunotherapeutic resistance by regulating cytotoxic T cells through upregulation of PD-L1 expression in cancer cells. YAP-mediated transcription of CXCL5, IL-6 and Csf1-3 promotes MDSC recruitment and macrophage infiltration.

**Figure 5:**
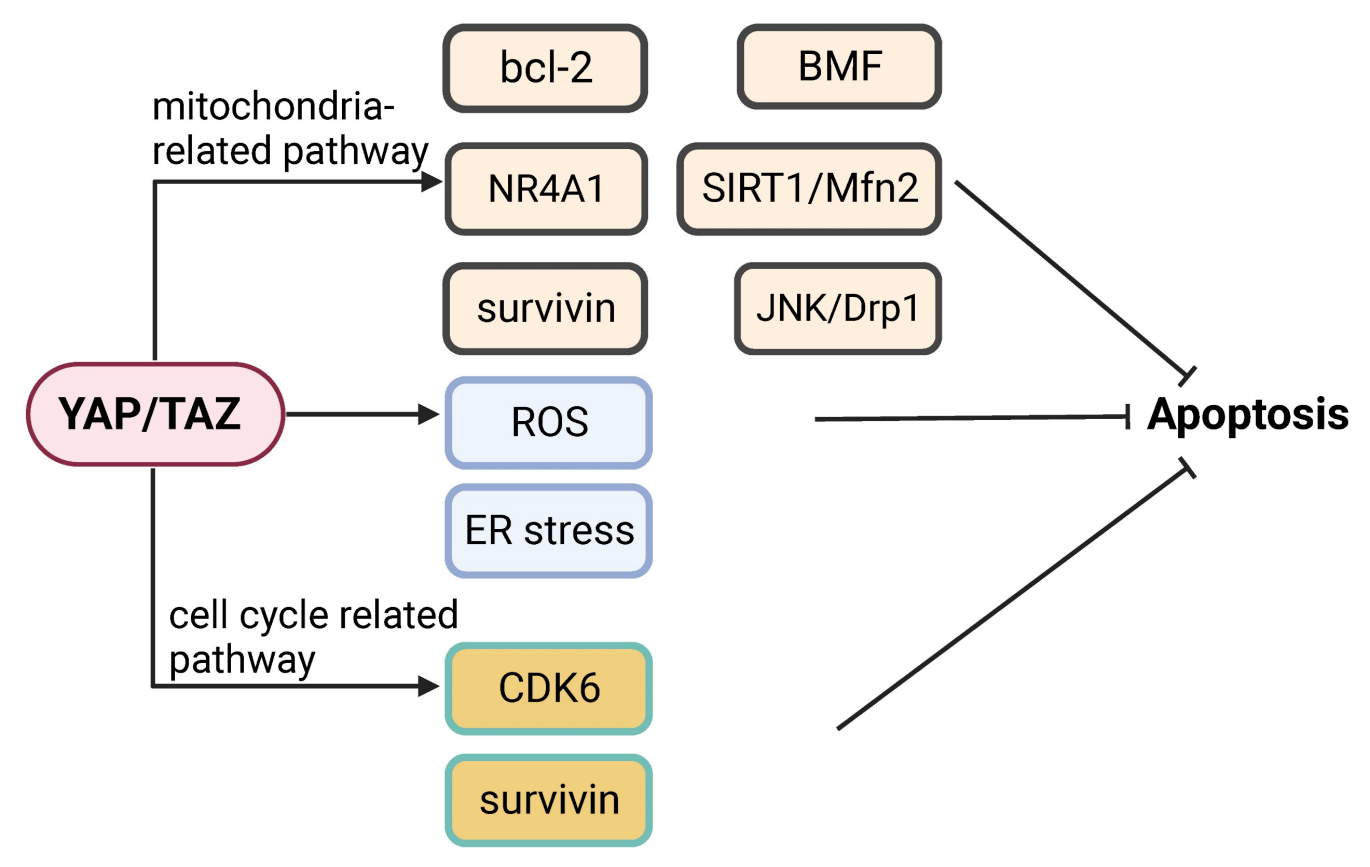
** YAP signaling in regulation of apoptosis.** YAP signaling can regulate apoptosis by modulating (1) Mitochondria-related events (e.g. bcl-2, BMF, NR4A1, SIRT1/Mfn2, survivin and JNK/Drp1); (2) ROS and ER stress responses; or (3) Mediators of the cell cycle, (e.g. CDK6 and survivin).

**Figure 6:**
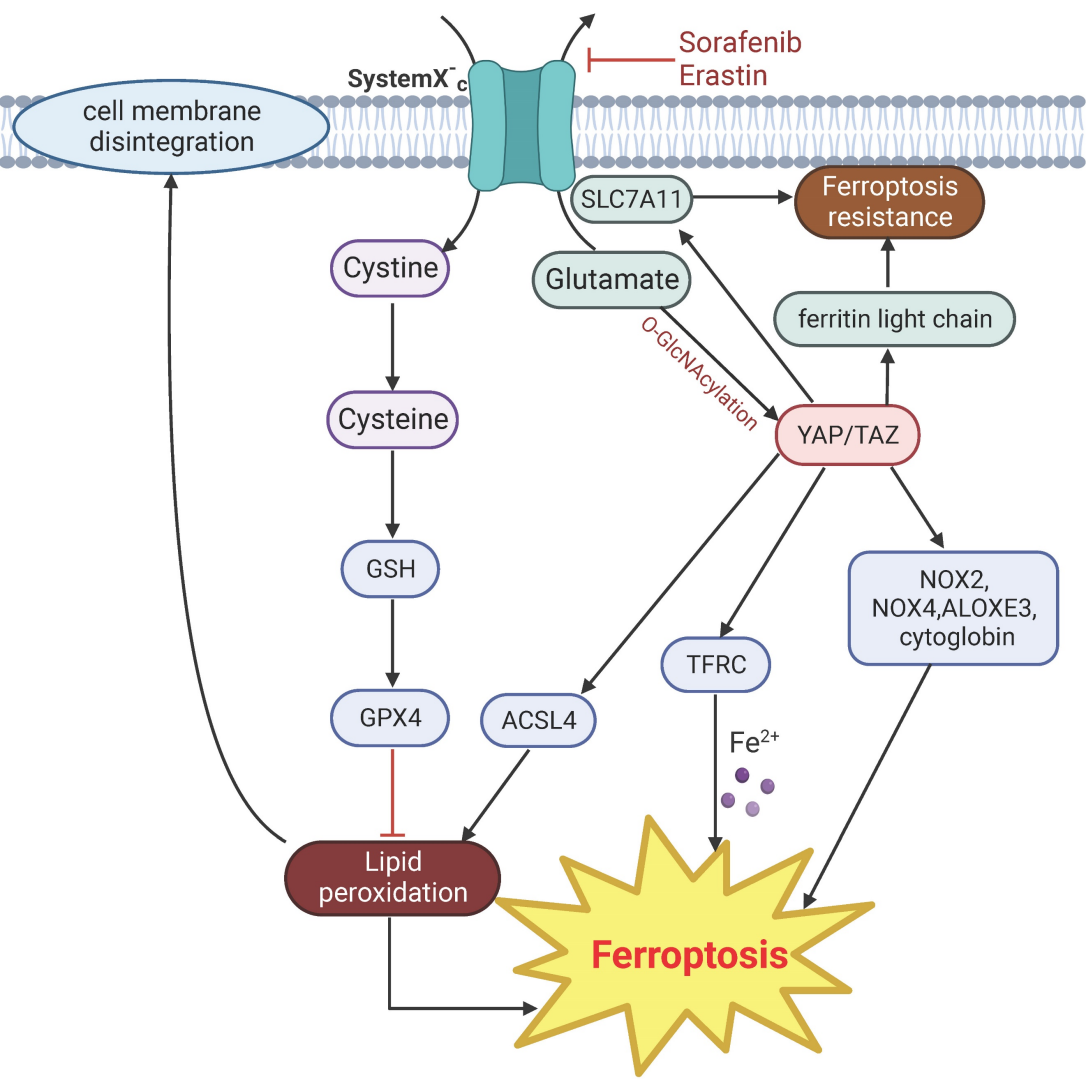
** YAP signaling related mechanisms in regulation of ferroptosis.** Drugs such as erastin and sorafenib directly target cystine transporter and trigger ferroptosis. Activation of YAP induce ferroptosis by upregulating the expression of ACSL4 and TRFC. Other regulators of YAP mediated ferroptosis include NOX2, NOX4, ALOXES3 and cytoglobin. Glutamate-induced O-GlcNAcylation of YAP inhibit ferroptosis. YAP signaling suppress ferroptosis by targeting SLC7A11 and ferritin light chain.

**Figure 7:**
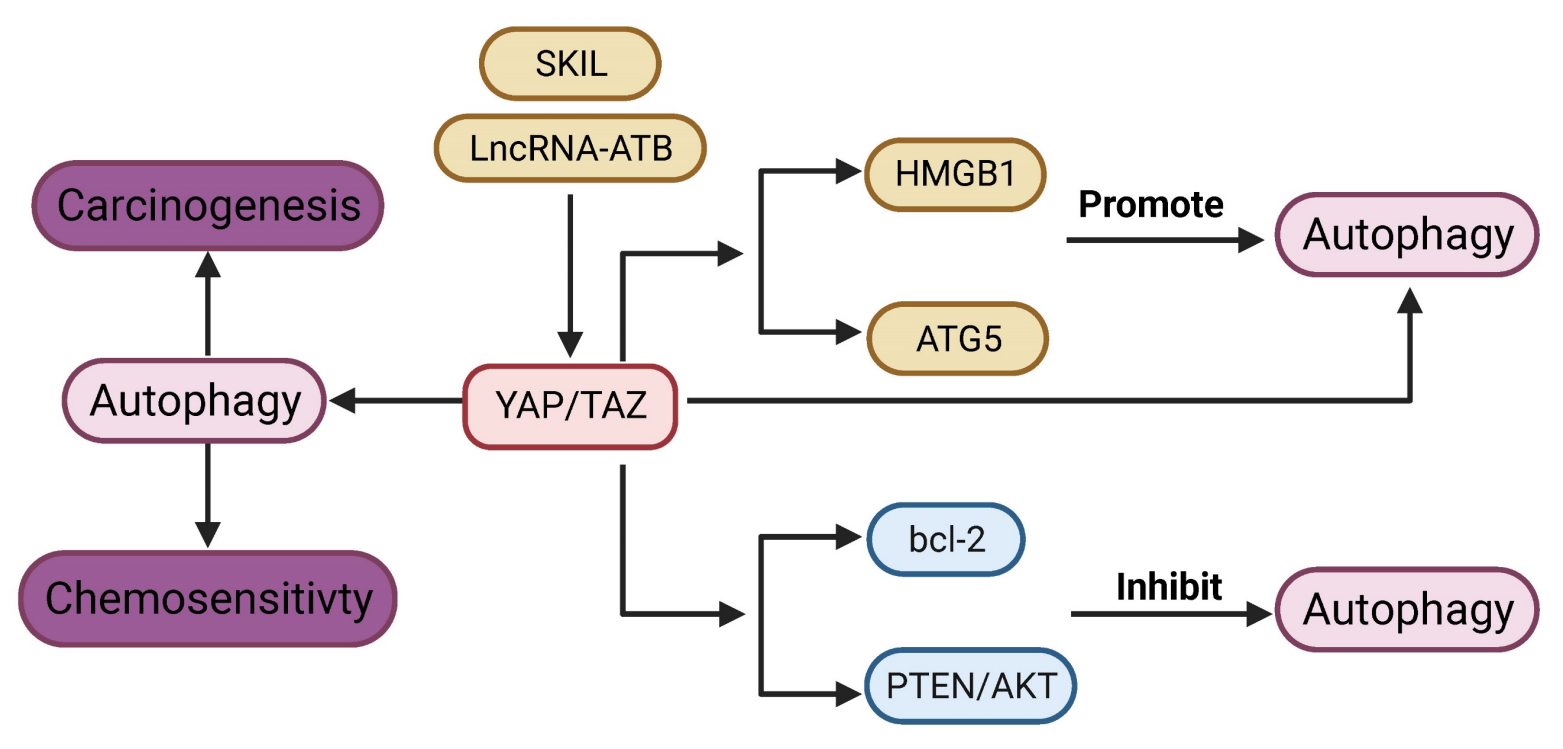
** Connection between YAP signaling and Autophagy.** lncRNA ATB and SKIL can promote autophagy via YAP signaling. YAP signaling promotes autophagy through regulation of HMGB1, ATG5. On the other hand, YAP signaling inhibits autophagy via bcl-2, and PTEN/AKT pathway. YAP signaling could regulate carcinogenesis and chemosensitivity via autophagy.

## Abbreviations

PCD: programmed cell death
YAP: yes-associated protein
TEAD: TEA domain family
MST: mammalian Ste20-like kinases
LATS: large tumor suppressor
MOB-1: Mps one binder kinase activator-1
KIBRA: KIdney and BRAin expressed protein
WWC1: WW and C2 domain containing 1
GPCR: G-protein coupled receptors
PTPN14: protein tyrosine phosphatase non-receptor type 14
SET1A: set domain containing 1A
HCC: hepatocellular carcinoma
SCLC: small cell lung cancer
HIF: hypoxia-inducible factor
TRIM11: tripartite motif-containing protein 11
CTGF: connective tissue growth factor
ABCB1: ATP binding cassette subfamily B member 1
CYR61: cysteine-rich angiogenic inducer 61
CDK: cyclin-dependent kinase
COX-2: cyclooxygenase 2
5-FU: 5-fluorouracil
RAR: retinoic acid receptors
RXR: retinoid X receptors
ALDH1A3: aldehyde dehydrogenase 1 family member A3
OCT4: octamer-binding transcription factor 4
EGFR: epidermal growth factor receptor
HER-2: human epidermal growth factor receptor 2
VEGFR: vascular endothelial growth factor receptor
TESK1: testis associated actin remodeling Kinase 1
TKI: tyrosine kinase inhibitor
EMT: epithelial-to-mesenchymal transition
NSCLC: non-small cell lung cancer
FOXM1: forkhead box protein M1
SAC: sacsin molecular chaperone
MEK: mitogen-activated protein kinase kinase
BET: bromodomain and extra-terminal motif
PD-1: Programmed death protein 1
PD-L1: programmed cell death ligand 1
CTLA-4: cytotoxic T-lymphocyte-associated antigen 4
TIGIT: T cell immunoglobulin and ITIM domain
MDSC: myeloid-derived suppressor cells
IL-6: Interleukin 6
CSF: colony stimulating factor
CXCL5: c-x-c motif chemokine ligand 5
ER: endoplasmic reticulum
PERK: protein kinase RNA-like ER kinase
EIF2a: eukaryotic translation initiation factor 2A
MAPK: mitogen-activated protein kinase
GPBAR: G protein-coupled bile acid receptor
JNK: c-Jun n-terminal kinase
NR4A1: nuclear receptor 4A1
KIF4A: kinesin family member 4A
BMF: Bcl-2-modifying factor
GPX4: glutathione peroxidase 4
GSH: glutathione
TFRC: transferrin receptor
ACSL4: acyl-CoA synthetase long chain family member 4
NOX: nicotinamide adenine dinucleotide phosphate oxidases
SLC7A11: solute carrier family 7 member 11
ALOXE3: Arachidonate Lipoxygenase 3
SUFU: suppressor of fused homolog
ANGPLT4: angiopoietin-like 4
EMP1: epithelial membrane protein 1
ATG: autophagy-associated proteins
ULK1: unc-51 like autophagy activating kinase
TNBC: triple-negative breast cancer
ANKRD1: ankyrin repeat domain 1
ERK: extracellular signal-regulated kinase
HMGB1: high mobility group box 1
UPR: unfolded protein response
RAC1: Rac family small GTPase 1
mTOR: mechanistic target of rapamycin kinase
PTEN: phosphatase and tensin homolog
GSDME: gasdermin E
MST1: macrophage stimulating 1
VP: verteporfin
